# Boron-doped diamond nanosheet volume-enriched screen-printed carbon electrodes: a platform for electroanalytical and impedimetric biosensor applications

**DOI:** 10.1007/s00604-023-05991-w

**Published:** 2023-09-22

**Authors:** Mateusz Ficek, Mateusz Cieślik, Monika Janik, Mateusz Brodowski, Mirosław Sawczak, Robert Bogdanowicz, Jacek Ryl

**Affiliations:** 1 Gdansk University of Technology, Narutowicza 11/12, 80-233 Gdańsk, Poland; 2 Department of Analytical Chemistry, University of Gdańsk, Wita Stwosza 63, 80-308 Gdańsk, Poland; 3 Institute of Microelectronics and Optoelectronics, Warsaw University of Technology, Koszykowa 75, 00-662 Warsaw, Poland; 4 Szewalski Institute of Fluid-Flow Machinery, Polish Academy of Sciences, Fiszera 14, Gdańsk, Poland

**Keywords:** Boron-doped diamond, Screen-printed electrodes, Electroanalysis, Pathogen detection, *Haemophilus influenzae*

## Abstract

This paper focuses on the development of a novel electrode based on boron-doped diamond nanosheet full-volume-enriched screen-printed carbon electrodes (BDDPE) for use as an impedimetric biosensor. Impedimetric biosensors offer high sensitivity and selectivity for virus detection, but their use as point-of-care devices is limited by the complexity of nanomaterials’ architecture and the receptor immobilisation procedures. The study presents a two-step modification process involving the electroreduction of diazonium salt at the BDDPE and the immobilisation of antibodies using zero-length cross-linkers for a selective impedimetric biosensor of *Haemophilus influenzae* (Hi). The incorporation of diamond nanosheets into BDDPE leads to enhanced charge transfer and electrochemical behaviour, demonstrating greatly improved electrochemically active surface area compared with unmodified screen-printed electrodes (by 44% and 10% on average for [Ru(NH_3_)_6_]Cl_2_ and K_3_[Fe(CN)_6_], respectively). The presented sensing system shows high specificity towards protein D in Hi bacteria, as confirmed by negative controls against potential interference from other pathogens, with an estimated tolerance limit for interference under 12%. The Hi limit of detection by electrochemical impedance spectroscopy was 1 CFU/mL (measured at − 0.13 V vs BDDPE pseudo-reference), which was achieved in under 10 min, including 5 min sample incubation in the presence of the analyte.

**Graphical abstract:**

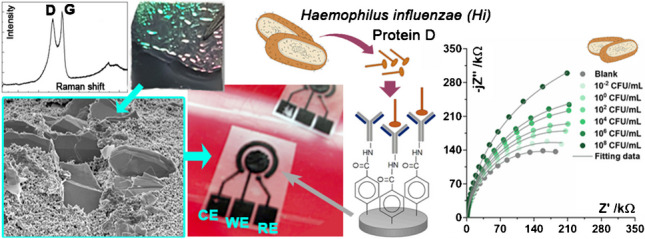

**Supplementary Information:**

The online version contains supplementary material available at 10.1007/s00604-023-05991-w.

## Introduction

High-quality boron-doped diamond surfaces are excellent electrochemical electrodes; nevertheless, their broad application is strongly limited by material cost, size scaling, and low specific surface area [1]. Conductive BDD powder (BDDP) was reported for the first time by Swain et al. in 2005 [2]. It was fabricated using insulating diamond powder as a substrate overgrown by BDD film in microwave plasma–assisted chemical vapour deposition (MPACVD). The mixture of BDDP with polymers or inks could be utilised in screen printing, yielding a BDD paste electrode. This approach makes it possible to overcome the high cost of standard BDD and its limited area scaling. This methodology was followed by works of Kondo et al*.* [3], revealing its efficiency in glucose [4] and dopamine detection [5], dental sterilisation by electrolysis [6], biopotential monitoring in plants [7], and bovine serum albumin detection [8], and mixed with insulating binder shows microelectrode-like characteristics [9]. Similar behaviour was observed for BDDP synthesised under high pressure and high temperature using B-doped graphite [10].

Next, Nantaphol et al. [11] showed the use of a BDD paste electrode (BDDPE) coupled with microfluidic paper-based analytical devices. Pastes were prepared from a mixture of BDD powder (Swain’s method) and mineral oil and can be easily stencil-printed into a variety of electrode geometries. They manifested that BDDPE could be applied for the detection of biological species (norepinephrine and serotonin) and heavy metals (Pb and Cd). BDDPEs exhibit a wider potential window and lower capacitive current than traditional carbon paste electrodes (CPE). Nevertheless, it should be mentioned that BDDP exhibits limited conductivity, revealing only inter-grain charge transfer along the BDD overlayer of the insulating diamond core. Furthermore, nanocrystalline diamond foils (typically 50 μm × 50 μm) grown by PACVD on quartz surfaces and delaminated due to thermal stress were reported by Seshan et al. [12]. Highly ordered diamond nanosheet arrays were manifested by Wang et al. [13], formed by in-plane epitaxy of diamond {111} planes at biased substrates. Freestanding crack-free BDD films can be fabricated by removing them from substrates through laser-cutting, followed by mechanical polishing processes [14]. Recently, thick BDD films (~ 500 μm in thickness) were peeled off a Mo substrate due to the larger difference in thermal expansion coefficients. They exhibited excellent super hydrophilicity after oxidisation treatment [15]. Fan et al. developed the transfer of boron-doped polycrystalline diamond onto a thin parylene-C for dopamine detection by lift-off by etching back a Si substrate [16]. Boron-doped detonation nanodiamond films were reported by Jackman et al. [17], revealing their semiconducting properties. This approach would open up new opportunities for nanodiamond-based electronic devices. Our prior preliminary work revealed an efficient method of fabrication of large-area, thin BDD nanosheets with interesting electronic transfer properties [18], along with an efficient tunnelling in diamond-on-graphene junction configuration [19]. Next, it was reported that 3D printouts made from commercially available graphene-doped polylactic acid (G-PLA) with surface-functionalised nanocrystalline boron-doped diamond foil (NDF) were found to be effective for detecting 2,4,6-trinitrotoluene (TNT) [20].

In the context of the increasing prevalence of antibiotic-resistant bacteria, it is especially important to develop effective detection methods. Early identification of the pathogen and the right drug to treat it can improve treatment outcomes and prevent the spread of antibiotic resistance [21]. One example of a bacterial infection that requires rapid and accurate identification is *Haemophilus influenzae* (Hi). This gram-negative bacterium is commonly found in the human respiratory tract and can cause a range of diseases, such as meningitis, pneumonia, middle ear infections, and sepsis. Traditional culture-based methods for detecting Hi can take up to 48 h, delaying diagnosis and appropriate treatment. Biochemical assays and molecular methods, such as polymerase chain reaction and sequencing, can offer faster detection times but often require specialised equipment and highly trained personnel [22, 23]. Amongst the many recognition mechanisms, tremendous attention in the last years is given to impedimetric biosensors. Impedance analysis offers critically high sensitivity, often unmatched by any other detection technique [24], with significant improvements in virus detection selectivity and specificity developed during the COVID-19 pandemic. These tempt with the potential of portability, low cost, real-time monitoring, and versatility [25]. On the other hand, the primary challenges in using impedimetric biosensors as point-of-care devices remain the complexity of the nanomaterials’ architecture and receptor immobilisation procedures as well as the non-specificity of the response mechanism [24]. The specificity of interaction can be improved by various antifouling mechanisms, such as bovine serum albumin deposition [26], generally increasing the level of surface complexity [27]. Using BDD and its derivatives as sensing platforms allows us to omit these steps. This is because BDD layers, in particular with oxygen-terminated surfaces, have excellent antifouling properties, being associated with hydrophilicity and consequently in reduced bacteria biofilm growth [28]. Both electrochemical characteristics and antifouling capability are affected by the doping level and the dominant faceted BDD, obtainable under controlled growth parameters [29]. Thus, the importance of the unique BDD property as a low biofouling electrode makes BDDPE highly promising for the rapid and sensitive detection of Hi and other pathogens. On the other hand, some of these architectures are complex, introducing problems of scaling the solution, material-wise [30].

In this study, we present a novel electrochemical electrode based on boron-doped diamond nanosheet full-volume-enriched screen-printed carbon electrodes (BDDPE) dedicated to electrocatalytic and electroanalytical purposes. The boron-doped diamond is known to be an effective electroanalytical platform, but assuring a high rate constant of the redox species depends on the uniform charge distribution in the electrochemically heterogeneous electrodes formed in BDDPE. These electrodes were synthesised using a one-step growth process through microwave plasma–assisted chemical vapour deposition (CVD), which involved the use of a gas mixture of H_2_:CH_4_:B_2_H_6_ on tantalum, and were isolated to form freestanding nanosheets that functioned as fillers. The incorporation of BDDPE led to enhanced charge transfer and electrochemical behaviour of carbon paste material in comparison to typical screen-printed electrodes (SPE), demonstrating improved kinetics and window. With the incorporation of diamond nanosheets into BDDPE, the adjacent electro-active surface areas start to overlap, and heavily overlapping diffusion fields act similarly to planar diffusion to a homogeneous surface. Additionally, the electrodes underwent a two-step modification process that involved the electroreduction of diazonium salt at the BDDPE and the immobilisation of antibodies using zero-length cross-linkers for selective impedimetric biosensors of Hi. The developed disposable biosensor exhibited a high level of sensitivity and selectivity towards protein D and Hi.

## Materials and methods

### Reagents and biomaterials

Phosphate-buffered saline (PBS), 4-aminobenzoic acid, N-hydroxysuccinimide (NHS), 1-ethyl-3-(3-dimethylaminopropyl)-carbodiimide (EDC), and PCR primers were purchased from Sigma-Aldrich (Germany). Potassium hexacyanoferrate (III), hexaammineruthenium (III), sodium nitrite, hydrochloric acid, and dipotassium phosphate monopotassium phosphate were purchased from ChemPur (Poland). An ExtractMe DNA Bacteria kit was purchased from Blirt (Poland), and a SensiFAST SYBR No-ROX kit was purchased from Bioline (UK). Phosphate buffer with different pH (5.68, 7.01, 7.99) was prepared by mixing the K_2_HPO_4_ and KH_2_PO_4_ in different ratios. All reagents were used without further purification. Aqueous solutions were prepared with the usage of double-distilled sterile water (ddH_2_O).

All biomaterials utilised in this manuscript are accurately described elsewhere [30]. Herein, we present them briefly. The monoclonal mouse antibodies were produced by the Nanobioengineering Laboratory at the Polish Center for Technology Development Ltd. through procedures described elsewhere [31], with slight changes. Recombinant protein D was isolated at the Institute of Biotechnology and Molecular Medicine following the standard procedures. The efficiency and quality of antibody production were verified with the ELISA test (data are not shown).

### Bacterium

*Hi* (strain 51907), *S. pyogenes* (strain 700294), and *S. pneumoniae* (strain 700674) were purchased from ATCC (US). *B. parapertussis* bacteria (strain 529), influenza A virus subtype H1N1, and *S. aureus* bacteria were provided from the Polish Collection of Microorganisms. A single colony of each bacterium was inoculated in BHI broth (Sigma-Aldrich), and cultured overnight at a temperature of 37 °C, and next, the culture was centrifuged and the cell pellet was resuspended in 1 mL of PBS buffer.

### Paste electrode (PE and BDDPE) fabrication

**Fig. 1:**
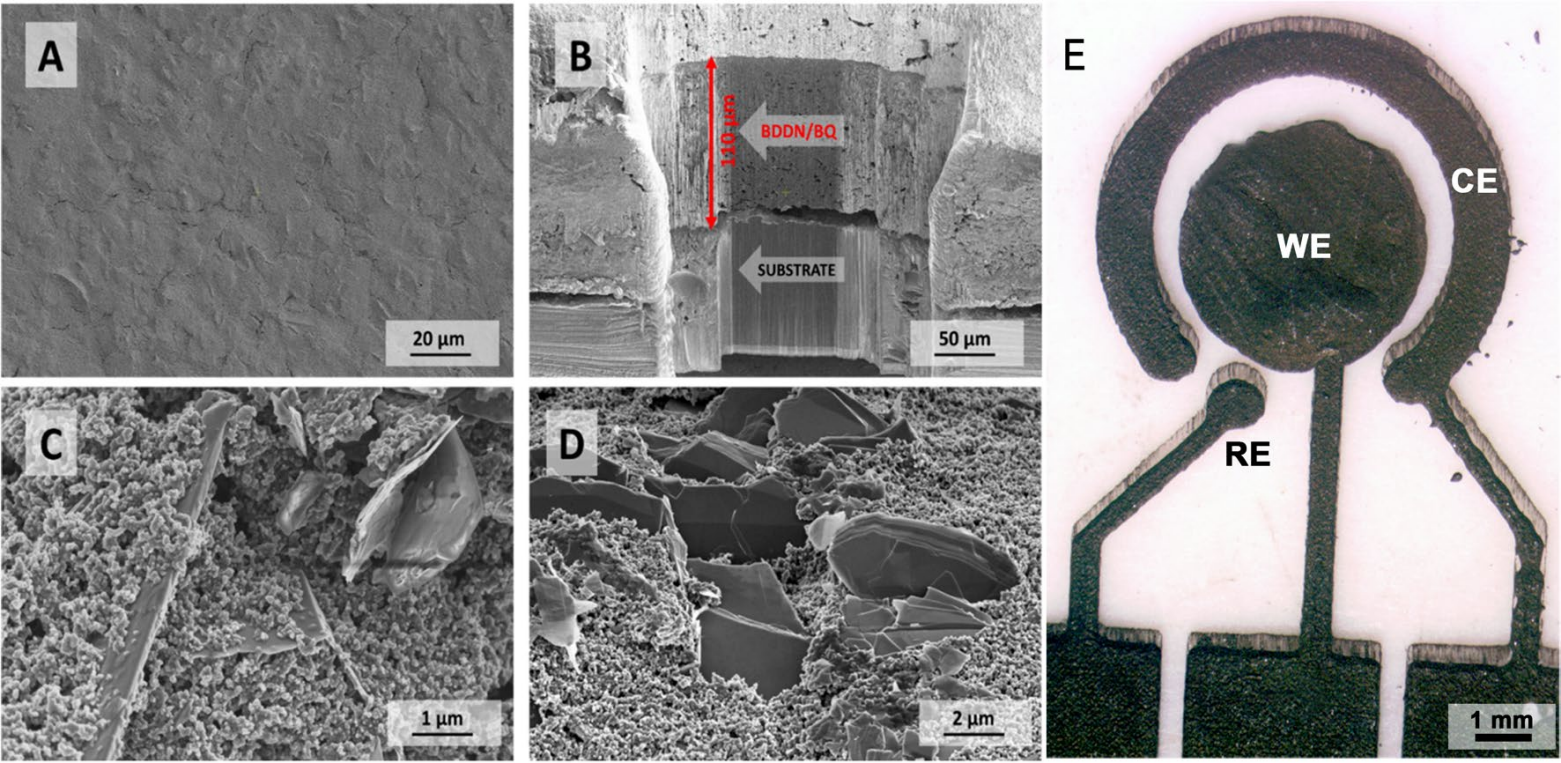
Topography of BDDPE displayed by SEM images: **A** top-view; **B** cross-section view; **C**, **D** zoomed-in top-view taken at various magnifications. **E** A BDDPE photograph with electrodes marked: WE, working electrode; CE, counter electrode; RE, reference electrode. The BDDPE-WE has a surface area of 0.152 cm.^2^

The boron-doped diamond foils were synthesised in an MWPACVD system (SEKI Technotron AX5400S), according to the details described in our previous work [18, 19]. The BDDPE was prepared according to the following 5-step procedure, as summarised in Table 1 and described in detail in SI file, Section S1. The BDDPE image was taken, as presented in Fig. 1E. Notably, the BDDPE-WE geometric surface area was 0.152 cm^2^.

**Table 1:** Five-step BDD paste electrode manufacturing procedure

Step	1. Shredding and grinding the foil	2. Carbon paste preparation	3. Dispersing the film with the paste	4. Forming BDDPE	5. Final curing
Equipment	Ceramic grinder	-	Mechanical stirrer	Semi-automatic screen printer	Laboratory dryer
Temperature	RT	RT	RT	RT	180 °C
Time	15 min	-	30 min	-*	15 min
Comment		DuPont BQ221	4% wt	325 mesh steel screen	In vacuum

^*^Squeegee speed 30 mm/s, squeegee pressure 1.2 kg

### Surface analysis techniques

An FEI Quanta FEG 250 Scanning Electron Microscope (SEM) with a 10-kV beam accelerating voltage with a SE-ETD detector (secondary electron—Everhart–Thornley detector) in high vacuum mode (pressure 10^−4^ Pa) was used to record the SEM images. The Raman spectra of the materials were recorded using a micro-Raman spectrometer (inVia, Renishaw, UK) equipped with a 514-nm argon-ion laser as an excitation source in combination with a 50 × objective (NA = 0.5) and a 10-μm confocal aperture. The X-Ray Photoelectron Spectroscopy (XPS) analysis was carried out using Escalab 250Xi (Thermo Fisher Scientific), operating with an AlKα X-ray source, spot diameter 250 µm, and with setup 30 eV pass energy through the hemispherical analyser. Low-energy electron and Ar^+^ ion bombardment were used for charge compensation.

### Electrochemical setup and evaluation

All electrochemical measurements were performed with a VMP-300 Biologic (France) potentiostat/galvanostat and the EC-Lab software, which was also used for modelling the EIS data. All measurements were performed in a three-electrode cell—the measurements that were focused on the stability and electrochemical performance of the sample (e.g. the open circuit potential (OCP) and electrolytic potential window measurements) were performed using a platinum wire as the counter electrode, Ag|AgCl|3 M KCl as the reference electrode, and the PE/BDDPE as the working electrode, and the volume of the electrolyte used was 1 mL. After the successful evaluation of the electrochemical parameters of the studied samples, the configuration of the samples was changed to that where all three electrodes consisted of PE or BDDPE, and the volume of the electrolyte was decreased to 200 µL which was used in the studies focused on electrochemical biosensing. All measurements were performed at room temperature, and all solutions were deoxygenated.

The OCP studies were conducted in a solution of 5 mM K_3_[Fe(CN)_6_] in 0.1 M PBS, with a pH of 7.39. Then, the OCP of the different elements of the samples (named -WE, -RE, and -CE, according to their intended role in the BDDPE) was recorded for 12 h. The measurements were performed three times for each sample and for multiple samples to obtain statistically significant data. During the experiments, the temperature was registered to ensure that it does not influence the potential. For redox kinetics studies, 5 mM K_3_[Fe(CN)_6_] or 1 mM [Ru(NH_3_)_6_]Cl_2_ was dissolved in PBS. The CVs was carried out at different polarisation rates in the range of 1 to 300 mV/s, with 10 cycles for each scan rate. The study was conducted in the range from − 0.4 to 1.0 V vs Ag/AgCl for the system with ferrocyanides and − 0.5 to 0.5 V vs Ag/AgCl for the system containing hexaammineruthenium. In the electrode stability tests, the CV polarisation range was − 2 to 2 V vs Ag/AgCl.

### BDDPE functionalisation for biosensing purposes

The functionalisation process has been presented previously in [30], generally including diazonium salt electrografting, followed by anti-protein D antibody anchoring using EDC/NHS chemistry. To properly consider material factors (mechanical strength, chemical, and electrochemical stability), some changes were made. The functionalisation details can be found in the SI file, Section S1.

Cyclic voltammetry (CV) and electrochemical impedance spectroscopy (EIS) were performed for bare (unmodified) BDDP. The CV (triplicate) was performed in the range from − 0.6 to 0.5 V vs BDDPE-RE pseudo-reference electrode, with a polarisation rate of 50 mV/s. The EIS frequency range was 10 kHz–1 Hz with 10 points per decade and the 10 mV peak-to-peak amplitude, measured at redox potential *E*_*F*_ (− 0.13 V vs BDDPE-RE pseudo-reference electrode). For all measurements involved in the investigation of using BDDPE as a biosensor, the solution was 1 mM K_3_[Fe(CN)_6_] dissolved in PBS. The electrolyte volume used was 200 µL.

## Results and discussion

### Physico-chemical and morphology study of the BDDPE

Figure 1 shows BDDPE SEM images at various magnifications. Higher magnifications reveal the clear, sharp shape of fragments of boron-doped diamond foil (Fig. 1C, D). These foil fragments are strongly bonded together with carbon paste. The size of individual diamond foil flakes is about 2 µm, whilst the conductive carbon particles within the used paste are up to 100 nm in size and stick together to form a continuous layer with small pores [32]. The paste fills in any gaps and imperfections on the surface of the diamond foil, improving the electrical contact between the foil and the composite matrix. In addition, the diamond foil facilitates the functionalisation of the surface compared to the paste itself. From the SEM images, it can be observed that the carbon paste does adhere quite tightly, creating connections between their fragments. The BDD foil is distributed evenly throughout the volume. The micrograph inset in Fig. 1A shows a cross-section of the entire BDDPE structure, which makes it possible to estimate the BDDPE thickness as 110 µm. Moreover, some foil pieces are protruding above the BDDPE surface, which usually leads to smaller steric hindrance effects when grafting large macromolecular compounds to the electrode surface, constituting an important advantage of such systems in electroanalysis [33, 34].

**Fig. 2:**
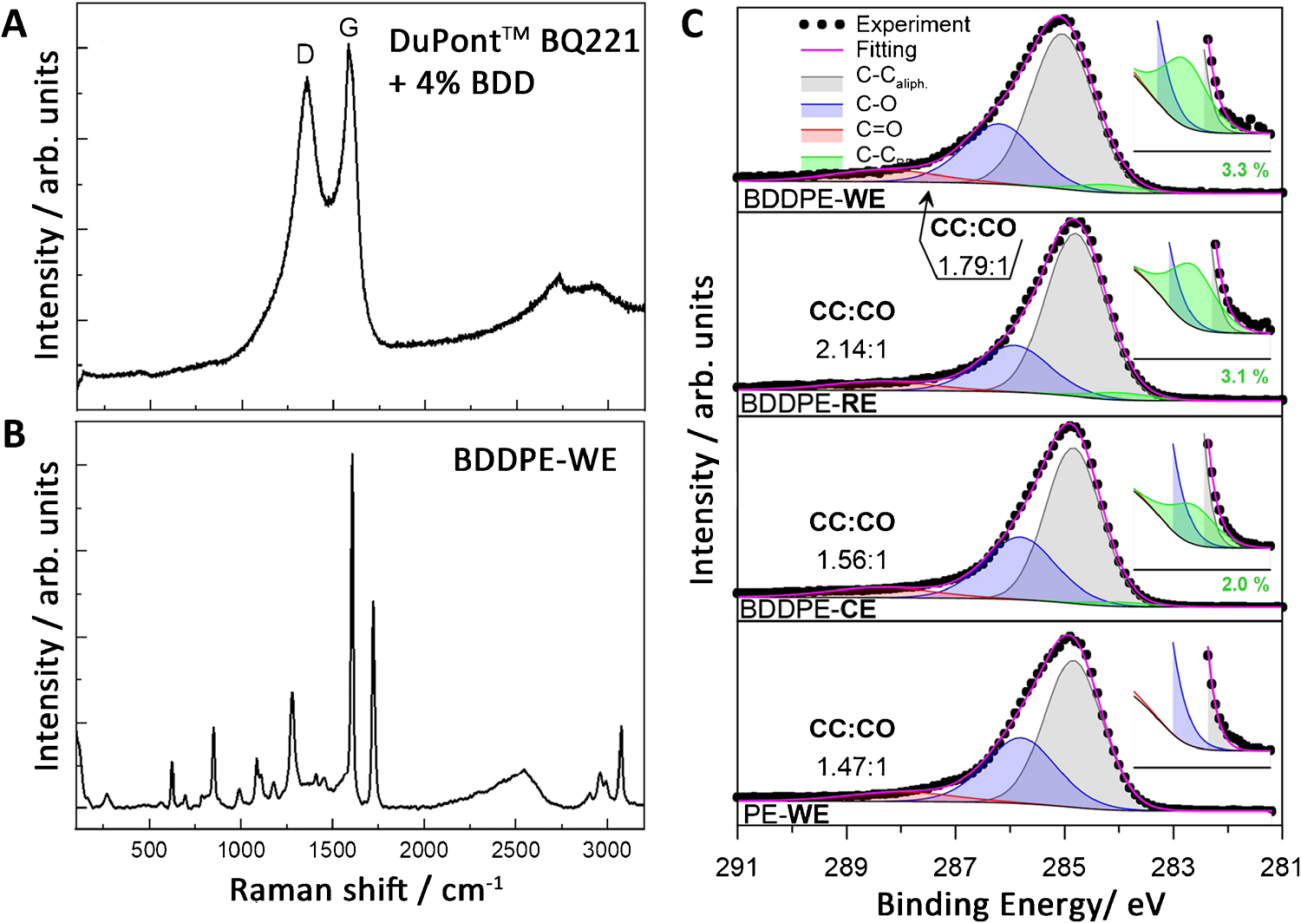
**A**, **B** Raman spectra: **A** carbon paste (DuPont BQ221) mixed with diamond foil, and **B** BDDPE deposited on PET foil. **C** High-resolution XPS analysis carried out in the C1s binding energy range, with proposed spectral deconvolution

The Raman spectrum of carbon paste mixed with diamond foil is presented in Fig. 2A. The spectrum is dominated by D and G bands centred at 1355 and 1585 cm^−1^, respectively, which can be assigned to sp^2^ carbon lattice structural defects and stretching vibrations of sp^2^ groups [35]. The *I*_*D*_/*I*_*G*_ ratio, which is a measure of the concentration of the defects in the sp^2^ structure [36], is estimated to be 0.93.

A small 4% addition of diamond foil is indistinguishable in the Raman spectrum, mainly because the characteristic bands of the BDD foil [18, 37] coincide with the Raman bands of the carbon filler used in the paste. In the Raman spectrum of thin carbon electrodes deposited on transparent inkjet printer film (Fig. 2B), mainly bands characteristic of polyethylene (polyethylene terephthalate) can be distinguished [38], and only a slight increase in the intensity of the Raman signal in the range of the D and G bands is observed, which can be explained by the low sensitivity of the Raman technique when examining thin films on a substrate that has pronounced Raman bands.

The surface analysis was expanded with the XPS analysis, performed for individual BDDPE and PE electrode areas. The spectra recorded in the C1s binding energy range reveal only small differences in the surface chemistry of the studied areas. The spectra show relatively complex characteristics and were deconvoluted using four components. Two major peaks at 284.9 add 285.9 eV are associated with the aliphatic C–C bonds and the oxidised C-O species in the paste electrode, with a third, much smaller component at 288.5 eV marking the presence of C = O species. The small peak located at 284.2 eV marks the presence of BDD in the studied paste [39]; its share was noted in each studied surface, reaching from 2 (for BDDPE-CE) to over 3% (BDDPE-WE and BDDPE-RE). Comparing the PE and BDDPE, one can recognise that the BDD foil incorporation does not significantly affect carbon chemistry; however, a slightly lower tendency to oxidise the electrode surface was observed for BDDPE (marked as the CC:CO components ratio).

Few studies have been published reporting the use of BDD in SPEs, as summarised in Table 2. Most of them focus on selective diamond growth in the required shape or the use of BDD powders. Amongst them, the BDDPE proposed by us offers the advantage of full screen-printability.

**Table 2:** Comparison of different protocols to fabricate screen-printed electrodes with boron-doped diamonds

BDD form	Matrix	Advantages	Challenges	Ref
Thin film	-	- Uniform BBD film - Standard BDD growth	- Selective growth of BDD - Substrate subjected to high temperature - Not a fully screen-printed electrode	[40]
Thin film	-	- Uniform BBD film - Standard BDD growth	- Selective growth of BDD - Substrate subjected to high temperature - Only RE was screen-printed	[41]
Thin film	Inkjet	- Standard BDD growth - Uniform BBD film	- Selective growth of BDD - Substrate subjected to high temperature - Only RE was screen-printed	[42]
Powder	Polyester resin	- Use of BDD powder - Scalability potential	- Low repeatability - No conductive polymer - Only surface modification	[43]
Powder	Polyester resin	- Use of BDD powder - Scalability potential	- Low repeatability - No conductive polymer - Only surface modification	[5]
Powder	Polyester resin	- Use of BDD powder - Scalability potential	- Low repeatability - No conductive polymer - Only surface modification	[44]
Powder	Laser-induced graphene	- Scalability potential - Fully screen-printed	- Substrate subjected to thermal shock - A small amount of BDD on the surface	[19]
Powder	Polylactic acid + carbon black	- 3D printout -Scalability potential	- BDD affects thermo-mechanical degradation - A small amount of BDD on the surface	[45]
Foil	Polylactic acid + carbon black	- 3D printout - Uniform BDD film	- Only surface modification - Printed substrate only	[20]
Foil	DuPont BQ221	- Fully screen-printed - Scalability potential	- A small amount of BDD on the surface	This work

### Evaluation of electrochemical properties of composite BDDPE material

**Fig. 3:**
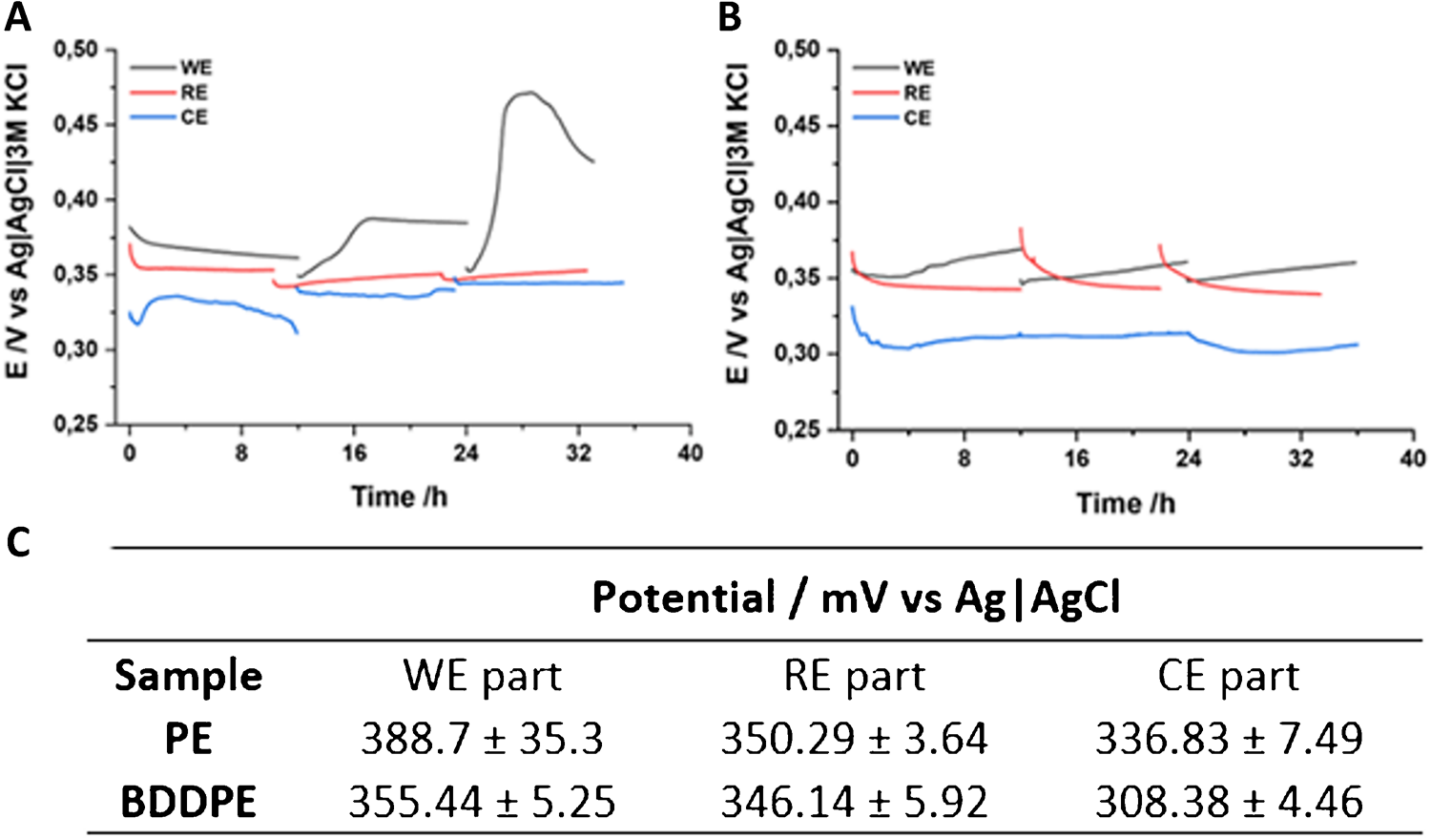
OCP values recorded for both studied pastes: **A** PE and **B** BDDPE, for 12 h and triplicated. **C** Statistical analysis of the OCP results, expressed as average potential ± standard deviation (*n* = 9 for PE and BDDPE)

The OCP constitutes an important measure of thermodynamic stability at the electrode/electrolyte interphase; if the OCP value is constant with a certain accuracy, one can assume electrode stability. It has a special role for the BDDPE-RE part, which will serve as a pseudo-reference electrode during polarisation studies. Figure 3 shows the results of such a measurement for PE (Fig. 3A) and BBDPE (Fig. 3B). It can be observed that the potential of the BDDPE-CE is slightly lower compared to that of BDDPE-WE and BDDPE-RE, regardless of the studied sample. This effect is most likely connected to a larger influence of the concentration cell, resulting directly from the experimental setup, with part of the substrate exposed next to the deposited paste. A significant discrepancy in the wettability of the substrate and the BDDPE could cause a concentration gradient of the redox compound K_3_[Fe(CN)_6_]. A higher concentration can be expected in the vicinity of the conductive paste, and therefore, a lower electrode potential is observed. Additionally, it can be seen that the potential of all BDDPE sample elements is lower compared to the corresponding parts of the PE sample. This may indicate increased activity of the diamond-enriched material. To summarise, the potential stability of the three-electrode system makes BDDPE possible to be used as a pseudo-reference electrode for polarisation studies.

When analysing the data presented in Fig. 3C, it can be seen that, for the most part, the potential of all parts maintains high stability—it does not exceed a deviation of 10 mV. The only exception is the PE-WE, which initially showed a high E increase during the second and the third measurements, which stabilised with time. Since the temperature changes during the measurement were very small, the potential changes are probably due to changes occurring at the electrode surface. Despite the low aggressiveness of the research environment, it is possible that the material began to hydrolyse or some adsorption layer started to form. This behaviour was not observed for the BDDPE sample, which may be due to the presence of hydrophobic diamond particles in the paste structure, which in turn hinders the hydrolysis process as it slows down water adsorption at the electrode surface.

Next, the electrolytic potential window value was established for both the PE and BDDPE samples. These data are presented in detail in the SI file, Fig. S1. The voltammograms testify small non-Faradaic currents in the polarisation range between − 0.5 and 1.5 V vs Ag|AgCl. Wide, delocalised reduction peaks were recognised at the cathodic scan below − 0.5 V vs Ag|AgCl for both studied samples, most likely originating from the decomposition of conductive composite paste. For electrochemically heterogeneous electrodes, the registered voltammograms depend on the kinetics of the individual electrode components [46]. Thus, the addition of BDD to the paste material increases the hydrogen evolution reaction overpotential due to the large electrolytic window provided by the BDD foil. Importantly, the BDDPE samples also overperformed the PE with increasing pH; however, on the other hand, the presence of BDD may have acted as a catalyst for the degradation of the PE.

The electrochemical activity of both materials was tested using CV with an increasing scan rate. Two types of redox pairs characterised by different electron transfer mechanisms were used in the research. The [Ru(NH_3_)_6_]Cl_2_ is known for its outer sphere electron transfer (OSET) whilst the more popular K_3_[Fe(CN)_6_] is typically the inner sphere (ISET). The main difference between these compounds is the necessity to create a temporary covalent bond between the electrode surface and the compound to exchange the electron in the latter case. As a result, it was possible to distinguish which stage of the electrode reaction limits the rate of the entire process [47]. The obtained CV curves are presented in the SI file, Figs. S2 and S3 and suggest an irreversible redox process in a wide range of applied polarisation scan rates.

The aforementioned data was used to estimate the electrochemically active surface area (EASA), *A*, by using the modified Randles–Sevcik equation for an irreversible, one-step, one-electron reaction [48]. The peak currents vs CV scan rate square root graphs are in the SI file, Fig. S4. The porous nature of the paste electrode produces a relatively high EASA, comparing the actual geometry of the WE element (0.152 cm^2^) with the PE surface available for the [Fe(CN)_6_]^3−/4−^ redox process, and similar regardless of the studied pH. Details of this analysis are presented in Table 3. Considering the electron transfer mechanism, [Fe(CN)_6_]^3−/4−^ is considered an ISET probe, meaning that the EASA is dependent on the local surface chemistry, and in particular on the presence of surface functional moieties with which the probe may form coordination bonds. Thus, studying the PE with an OSET redox probe, [Ru(NH_3_)_6_]^2+/3+^, leads to an approx. 30% increase in the measured EASA.

**Table 3:** Electrochemically active surface area (EASA, in cm^2^) for PE and BDDPE electrodes

	PE		BDDPE	
pH	[Ru(NH_3_)_6_]^2+/3+^	[Fe(CN)_6_]^3−/4−^	[Ru(NH_3_)_6_]^2+/3+^	[Fe(CN)_6_]^3−/4−^
5.7	0.721	0.505	1.040	0.551
7.0	0.764	0.531	1.150	0.551
8.0	0.721	0.467	0.991	0.558

It should be emphasised that the presence of the BDD foil within the electrode leads to a significant, nearly 8 × enhancement in the EASA available for the [Ru(NH_3_)_6_]^2+/3+^, a tremendous 67% increase compared to the PE. This effect is less notable and only approx. 10% for [Fe(CN)_6_]^3−/4−^, which is most likely connected with the surface chemistry of the BDD foils, composed of oxygen-terminated polycrystalline boron-doped diamond [49]. Their detailed electrochemical performance will depend on the dopant density but also the side (top or bottom) exposed to the electrolyte [37]. Regardless of these variables, the EASA measured remains steady in the whole studied pH range. The standard deviation for the surface chemistry sensitive [Fe(CN)_6_]^3−/4−^ probe does not exceed 1% from the average value in the case of BDDPE (6% for PE). The previously observed tremendous increase in the EASA by BDD foil incorporation most likely originated from several coexisting factors. The high-magnification SEM micrographs revealed the presence of connections in-between foil fragments and the surrounding paste, which expands the BDDPE microporosity. Furthermore, an additional geometry factor is provided by protruding pieces of foil.

The above conclusions find representation in the kinetics of the charge transfer process. Taking data from the SI file, Figs. S2 and S3 and the 50 mV/s scan rate as an example, the current registered at the [Ru(NH_3_)_6_]^2+^ oxidation peak for BDDPE surpasses that registered at PE by 50%, proving the superior EASA of the former electrode. Similarly, the mechanism of the redox process did not change significantly, noted by similarities in the anodic-to-cathodic peak separation and current rate, *i*_*A*_:*i*_*C*_, which is approx. 0.7:1 for [Ru(NH_3_)_6_]^2+/3+^ and 0.95:1 for [Fe(CN)_6_]^3−/4−^, regardless of the electrode type.

### Validation of the BDDPE system as a biosensor platform

To determine the biosensing capabilities of BDDPE, Hi was chosen as a model biological target. The structure of Hi is characterised by several features. The first is a capsule. Some strains of Hi are encapsulated, meaning they have a polysaccharide capsule surrounding the cell wall. The capsule is one of the virulence factors that helps the bacterium evade the host immune system [50]. The next feature is its pili, which are hair-like structures that protrude from the surface of the bacterium. They are involved in adhesion to host cells and the uptake of genetic material [51]. Hi also produces a variety of proteins, including virulence factors such as protein D [52]. These proteins are often surface-exposed and play important roles in the bacterium’s interactions with the host [53]. All the mentioned features are important targets for both the host immune response and, at the same time, the development of sensing systems that can specifically recognise Hi infections [54, 55]. In our work, we will focus on protein D, which is highly conserved amongst different strains of the bacterium, and which has been proven to be a relevant target for its diagnostics. To ensure the specificity of our sensing system, the BDDPE’s surface was functionalised, leading to uniform BDDPE coverage with immobilised anti-protein D antibodies. It is important to mention that, in comparison to PE, BDDPE has numerous reactive sites on its surface that can increase the density of the functionalisation sites, thereby increasing the efficiency of surface modification [56, 57].

**Fig. 4:**
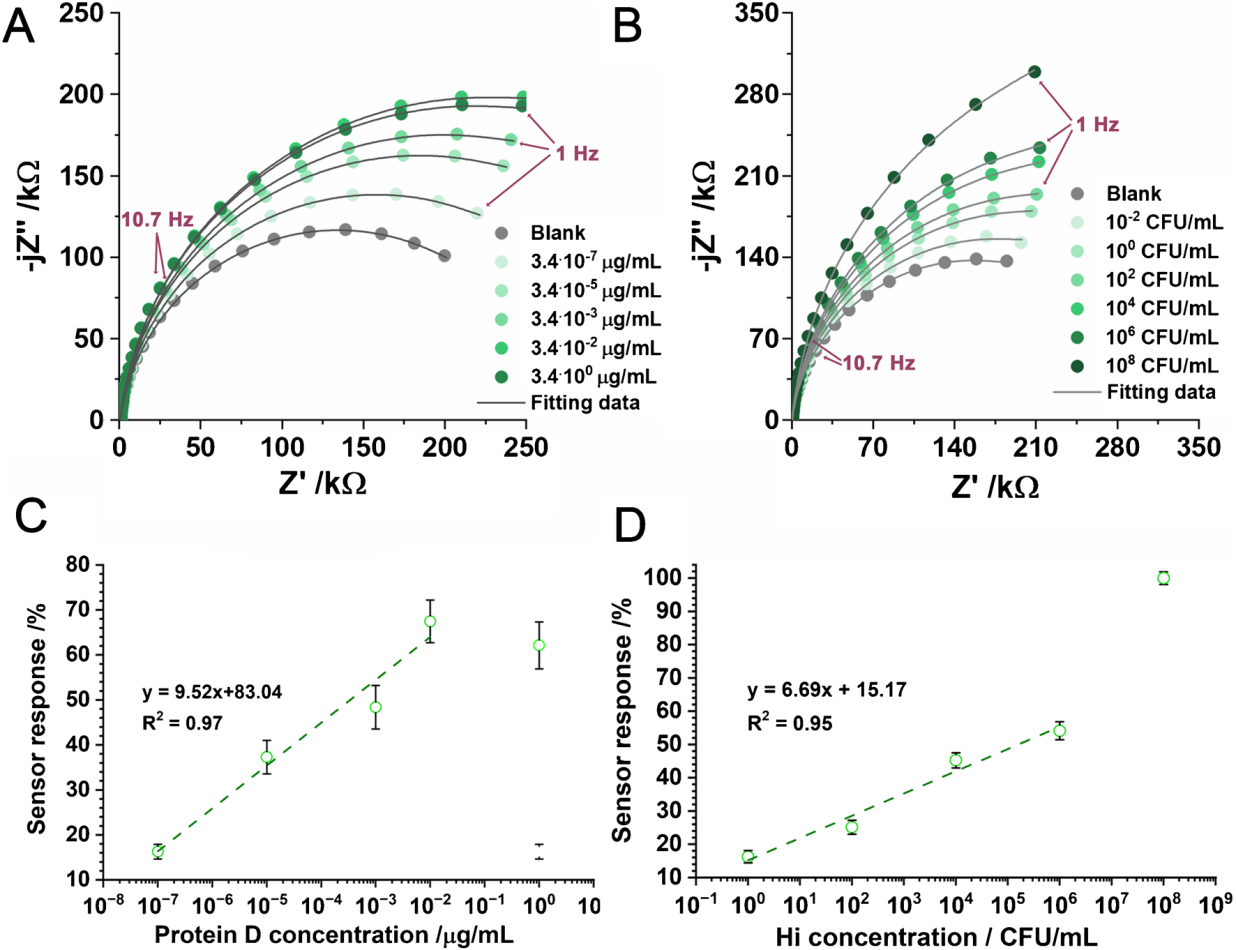
**A**, **C** Protein D, and **B**, **D** Hi detection using receptor-functionalised BDDPE. **A**, **B** EIS spectra in Nyquist projection at different analyte concentrations. Frequency range 10 kHz–1 Hz, measured at − 0.13 V vs BDDPE-RE pseudo-reference electrode (*E*_*F*_) after 5 min incubation. **C**, **D** Calibration curves of sensor response, estimated with Eq. (2)

After the surface modification, the sensor was checked towards the selected target—protein D. Protein D is a relatively large lipoprotein with a molecular weight of approximately 43 kDa and contains several antigenic epitopes that can be recognised by antibodies [58]. Because of the many acidic amino acid residues, it generally has a net negative charge at physiological pH; therefore, independently of the binding site and its conformation on the sensor’s surface, it should affect the sensor’s response similarly. Figure 4A shows the impedance spectra recorded at the BDDPE-WE in the system using BDDPE as all three electrodes, at different protein D concentrations in the range between 3.4 × 10^−7^ and 3.4 µg/mL.

Appropriate analysis of the recorded impedance data utilised the fitting procedure with an electric equivalent circuit (EEC), pre-selected in such a way as to represent the electric processes at the electrode surface. The EEC is composed of the parallel connection of the charge transfer resistance (*R*_CT_) and electric double layer capacitance (*C*_DL_), modelled by a constant phase element (CPE) to include the electric heterogeneity of the BDDPE. The impedance of the CPE is described by Eq. (1)

1 $${Z}_{\mathrm{CPE}}=\frac{1}{Q{\left(j\omega \right)}^{n}}$$

where *w* is the angular frequency, *Q* represents the *quasi*-capacitance of the electric double layer, and CPE exponent *n* is the heterogeneity factor. For *n* = 1, the CPE represents an ideal capacitor with capacitance *Q*, whilst the lower the *n*, the higher the frequency dispersion of capacitance introduced by the heterogeneous surface. We also estimated the effective *C*_DL_ using the surface distribution model proposed by Hirschorn et al. [59]. Increasing the concentrations of protein D, and therefore binding more protein to the anti-protein D antibodies immobilised on the electrode’s surface, results in a gradual and effective *R*_CT_ increase assisted by a slight drop of the *quasi*-capacitance *Q*. The detailed results of the analysis are summarised in Table 4.

**Table 4:** EIS results calculated with the above-proposed EEC

		CPE / µFs^*n*−1^	*n* / -	*R*_CT_ / kΩ	*C*_DL_ / µF
Protein D µg/mL	Blank	1.63	0.91	267.9	0.82
	3.4۰10^−7^	1.59	0.92	314.8	0.85
	3.4۰10^−5^	1.57	0.92	367.8	0.87
	3.4۰10^−3^	1.59	0.92	397.5	0.86
	3.4۰10^−2^	1.59	0.92	448.6	0.87
	3.4۰10^0^	1.58	0.93	434.3	0.89
*H. influenzae* CFU/mL	Blank	1.67	0.92	346.6	0.79
	1	1.95	0.92	402.9	1.10
	10^2^	1.91	0.93	433.7	1.11
	10^4^	1.90	0.93	503.4	1.06
	10^6^	1.88	0.93	541.1	1.05
	10^8^	1.82	0.94	746.0	1.09

The most pronounced *R*_CT_ increase was reported for the first two protein D concentrations, whilst for the highest ones, the trend of changes slowly saturates. Saturation of all available binding places results in the loss of linearity at the highest protein D concentration of 3.4 µg/mL. Figure 4C presents the calibration curve, which was constructed based on the obtained values. For improved readability and comparison of different immunosensors and samples, the relative change in *R*_CT_ (labelled as sensor response) was calculated according to Eq. (2):

2 $$\mathrm{Sensor response}=\frac{{{R}^{\mathrm{test}}}_{\mathrm{CT}}- {{R}^{\mathrm{blank}}}_{\mathrm{CT}}}{{{R}^{\mathrm{blank}}}_{\mathrm{CT}}} \bullet 100\mathrm{ \%}$$

where *R*^blank^_CT_ is the *R*_CT_ in the absence of the analyte (reference measurement), and *R*^test^_CT_ is the *R*_CT_ when the electrode is subjected to the analyte at a given concentration. In this way, the sensor response can be interpreted as the ratio of the modified surface area to the sensor’s total available surface area and, therefore, can eliminate differences between results that are caused by slight differences in the sensors’ surface initial states. The sensor response linearity range covers protein D concentrations ranging from 10^−7^ to 10^−2^ µg/mL, and the relatively small error bars indicate high repeatability of measurements. One should also note that the CPE exponent *n* slowly increases with the analyte concentration, a phenomenon which is explicable by the increase in electric homogeneity at the surface upon the increase of the BDDPE surface coverage by the adsorbed layer formed by protein D covalently bound to the receptor.

Effective detection of protein D enabled the use of the reported sensing system for the analysis of more complex samples containing whole bacteria cells. Whole bacteria detection is more complex than single-protein identification for many reasons. As mentioned at the beginning of this section, the structure of Hi is characterised by several features, including a polysaccharide capsule and pili. The analysed strain is a type B strain, and it is known to produce both. Their presence can affect the accuracy and sensitivity of any label-free detection method. They can cause non-specific binding to the sensor’s surface, leading to false-positive results. Additionally, they can physically block access to the protein D localised on the bacteria cell wall or cause steric hindrance, which prevents efficient antibody binding. However, the exact extent to which the pili and polysaccharide capsules interfere with the binding of antibodies to protein D can depend on several factors, such as their density and distribution on the bacterial surface, the orientation of the protein D molecules, and the affinity and specificity of the antibodies used as receptors. In some cases, the presence of for example pili can enhance the binding of antibodies to protein D, as pili can act as a scaffold or a platform for the presentation of multiple copies of the protein D antigen [51].

Figure 4B shows impedance spectra recorded for subsequent additions of the Hi solution with increasing concentrations starting from 10^−2^ to 10^8^ CFU/mL. The measurement procedure was similar to protein D. Here, the highest concentrations affect the spectra the most, and at 10^8^ CFU/mL, the sensor response does not follow a linear function with the Hi concentration, which might suggest the appearance of non-specific, weak vdW interactions (see Fig. 4D). It needs to be noted that, except for the abovementioned functions, pili and polysaccharides are also involved in bacterial aggregation and biofilm formation. Therefore, instead of a single bacteria layer on the sensor’s surface, we may also expect a multilayer during the incubation of the sensor with higher bacteria concentrations, which will surely further affect the obtained signal. Nevertheless, the direct detection of Hi was possible by studying the charge transfer resistance response, with a limit of detection (LOD) as low as 1 CFU/mL; however, similar to protein D, the changes in *C*_DL_ provide no quantitative information. The LOD was determined using the 3*σ*/*m* formula, where *σ* is the standard deviation of the intercept and *m* is the slope of the calibration plot [60]. Table 5 contains the comparison of LOD by different, recently reported electrochemical biosensors LOD.

**Table 5:** The comparison of the electrochemical biosensors for detection of protein D and Hi at different electrodes

Substrate	Detection technique	Limit of detection	Ref
Boron-doped carbon nanowalls	Electrochemical impedance spectroscopy	5.20 10^2^ CFU/mL	[30]
Black phosphorus	Electrochemical impedance spectroscopy	5.82 µg/mL	[61]
Zn-based MOF/CMC Au NP	Differential pulse voltammetry	1.48 fM	[62]
Au@Ag core–shell/GQDs ink	Chronoamperometry	LLOQ = 100 zM	[63]
Ag-DPA-GQD ink/Cysa-Au NPs	Square wave voltammetry	LLOQ = 1 zM	[64]
Reduced graphene oxide	Chronoamperometry	0.5 PFU/mL	[65]
NiCr-layered double hydroxide at Au	Differential pulse voltammetry	6.14 fM	[66]
CTAB/AgNPs	Spectrofluorometric and UV/Vis spectrophotometric	LLOQ = 1zM	[67]
-	Duplex quantitative PCR	0.0125 pg	[68]
-	Quantitative real-time PCR	104 DNA copies/mL	[69]
BBDPE	Electrochemical impedance spectroscopy	1 CFU/mL	This work

The BDDPEs were evaluated for 2 weeks post-fabrication. These electrodes were stored at room temperature, shielded from light exposure. Impressively, the stored electrodes exhibited robust stability in their analytical performance, showing no noticeable signal deterioration. The sensor’s longevity depends on receptor quality (e.g. lyophilised antibodies) and preparation. Under ideal storage (2–8°Ct), the sensor should last up to a year [70]. To gauge the electrode reproducibility, we assessed their response to protein D in varying concentrations of Hi bacteria. Four separate test electrodes were fabricated for each target. The results indicated high reproducibility, with a relative standard deviation below 4% for Hi and 3% for protein D. This underscores the high consistency of the developed electrode material. Moreover, the average standard deviation error remained below 2% across the concentration ranges examined. The test with a disposable BDDPE costs about 15 Euro cents (counting the cost of materials per test), and was designed for onetime use in diagnostics.

**Fig. 5:**
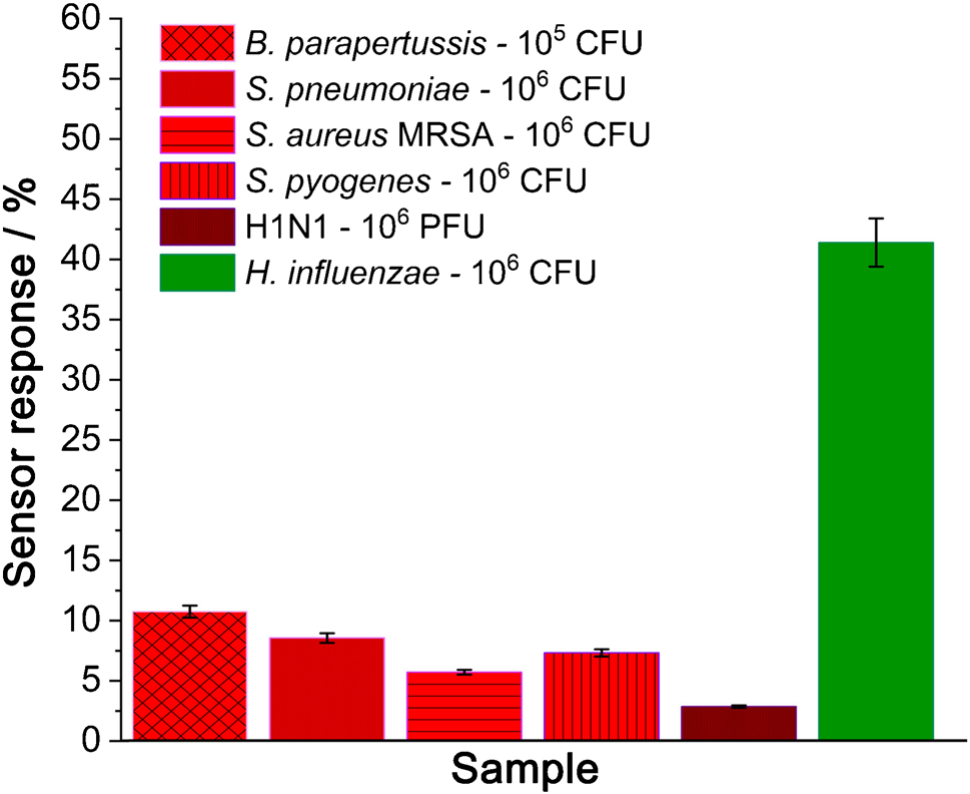
Results of cross-reactivity tests: Sensor response recorded for different pathogens, i.e. *S. parapertussis*, *S. pneumoniae*, *S. pyogenes*, *S. aureus*, Hi, and H1N1 virus at a given concentration. The EIS frequency range was 10 kHz–1 Hz, measured at − 0.13 V vs BDDPE-RE pseudo-reference electrode (*E*_*F*_) after 5 min of incubation

Finally, to test the specificity of the presented sensor and to be sure that even the smallest changes were induced by the change in the composition of the biolayer on the sensor’s surface, a series of negative controls were performed. The following pathogens, namely *B. parapertussis*, *S. pyogenes*, and *S. aureus* bacteria, as well as H1N1 virus, were identified as potentially interfering with the targeted Hi. All of them can cause respiratory infections [71, 72]; moreover, they all present a variety of surface proteins that could non-specifically bound to the chosen bioreceptor. To receive comparable results, the concentrations of the tested pathogen samples were kept in the same order of magnitude. Every sample was tested on a separate electrode. Figure 5 presents the obtained results recorded after 5 min of incubation of the sensor with each sample, whilst the detailed data are given in the SI file, Fig. S5 and Table S1.

The recorded changes ranged from 2.84% (H1N1) to 10.73% (*B. parapertussis*). Thus, the sensor’s tolerance limit was set at 12%. The most apparent non-specific interactions observed in the case of *B. parapertussis*, *S. pyogenes*, and *S. pneumoniae* may stem from two different reasons. Firstly, non-specific interactions occurring between anti-protein D antibodies and these bacterial strains might be considered. However, this is unlikely due to the significant variations in bacterial morphology and surface proteins across the species. The second cause for the observed non-specific signals could be attributed to the morphology of the electrode surface. The physical structure and characteristics of the electrode surface could potentially facilitate the adhesion of these well-developed microorganisms, resulting in non-specific interactions. However, considering the values obtained for the positive control (41.38%), it can be stated that none of the tested pathogens produces a substantial impedance increase, which confirms the high specificity of the presented sensing system towards protein D in Hi bacteria.

## Conclusions

This study introduces a novel electrochemical biosensor electrode based on boron-doped diamond nanosheet full-volume-enriched screen-printed carbon electrodes (BDDPE) for electrocatalytic and electroanalytical purposes. The incorporation of diamond nanosheets into BDDPE led to a greatly improved electrochemically active surface area and enhanced electrolytic window. The BDDPEs were successfully tested in the pH range of 5.7 to 8.0 and revealed stable potential even during a 36-h test. The material is a good candidate for the pseudo-reference electrode, which constitutes an advantage considering that typically a silver-based pseudo-reference electrode is utilised. However, the small amount of BDD on the BDDPE surface may limit its environmental resistance.

The developed biosensor exhibited a high level of sensitivity and selectivity towards protein D and Hi, with a Hi limit of detection as low as 1 CFU/mL and a calibration curve linearity range of up to 10^6^ CFU/mL. It should be highlighted that the impedimetric recognition, including electrode incubation, is done in under 10 min. To ensure the specificity of the presented sensor, a series of negative controls were performed. The results showed that none of the tested pathogens produced a substantial impedance increase, confirming the high specificity of the presented sensing system towards protein D in Hi bacteria. Most notably, the above-determined performance was obtained whilst not utilising any common antifouling mechanisms. Overall, this study presents a promising platform for electrocatalytic and electroanalytical purposes, with potential applications for rapid biological and medical disposable sensors.

### Supplementary Information

Below is the link to the electronic supplementary material.

Supplementary file1 (DOCX 2134 KB)

## Data Availability

The data that support the findings of this study are available from the corresponding author, [J.R. and R.B], upon request.
