# Supplementary material for: Boron-doped diamond nanosheet volume-enriched screen-printed carbon electrodes: a platform for electroanalytical and impedimetric biosensor applications
Source: Mikrochim Acta. 2023 Sep 22;190(10):410. doi: 10.1007/s00604-023-05991-w (PMC10516795; doi:10.1007/s00604-023-05991-w)
Supplement: Supplementary file 1 — Supplementary file1 (DOCX 2134 KB) [file 604_2023_5991_MOESM1_ESM.docx]

Supplementary Information file

for

**Boron-Doped Diamond Nanosheet Volume-Enriched Screen-Printed Carbon Electrodes: A Platform for Electroanalytical and Impedimetric Biosensor Applications**

Mateusz Ficek^1^, Mateusz Cieślik^1,2^, Monika Janik^1,3^, Mateusz Brodowski^1^, Mirosław Sawczak^4^, Robert Bogdanowicz^1,*^ and Jacek Ryl^1,*^

^1^ Gdansk University of Technology, Narutowicza 11/12, 80-233 Gdansk, Poland

^2^ Department of Analytic Chemistry, University of Gdańsk, Wita Stwosza 63, 80-308 Gdańsk, Poland

^3^ Institute of Microelectronics and Optoelectronics, Warsaw University of Technology, Koszykowa 75, 00-662, Warszawa, Poland

## ^4^ Szewalski Institute of Fluid-Flow Machinery, Polish Academy of Sciences, Fiszera 14, Gdansk, Poland

Corresponding author: [jacek.ryl@pg.edu.pl](mailto:jacek.ryl@pg.edu.pl) / [robbogda@pg.edu.pl](mailto:robbogda@pg.edu.pl)

**S1. Experimental details**

**S.1.1. BDDPE fabrication details**

BDD foils were synthesized in the MWPACVD system on mirror-polished tantalum foils (Sigma-Aldrich Chemie, 0.025 mm thick, 99.9+% metal basis). Substrates were seeded ultrasonically in an aqueous suspension containing nanodiamond powder (4–7 nm in size). The temperature of the heated graphite stage was kept at 500 °C during the deposition process. The CH_4_:H_2_ molar ratio of the mixture in this study was set at 1% of gas volume at 300 sccm total flow rate. The doping level of boron in the gas phase, expressed as the [B]/[C] ratio, was 10 000 ppm. The growth time was 300 min.

The initially produced boron-doped diamond foils were mechanically removed from the tantalum substrate and were then placed in the ceramic grinder for 15 minutes. The resulting ground diamond foil was added to a commercial carbon paste (DuPont BQ221, USA) and mixed until a homogeneous mass was obtained. The ratio of diamond foil to paste was 4%. Forming BDDPE consists of its deposition on a transparent film for inkjet printers by screen printing (Polyethylene terephthalate). Three regions are distinguished in the BDDPE construction: working electrode (BDDPE-WE), reference electrode (BDDPE-RE), and counter electrode (BDDPE-CE). Steel screen with 325 mesh, squeegee speed - 30 mm/s, and squeegee pressure 1.2 kg were used to produce. The electrode was finally dried and heated at 180°C for 15 minutes.

**S.1.2. BDDPE functionalization details**

Firstly, before the functionalization, the BDDPE has been gently rinsed with demineralized water to remove any possible contamination and dried with a stream of argon. After that, the BDDPE was placed in an electrochemical cell (diameter of 7 mm).

After the successful first step of modification, the solution of 34.5 mg EDC and 20.9 mg NHS was prepared. The 40 µL of 100-times diluted antibodies stock in PBS was added to the mixture of NHS and EDC. Then 50 µL of a solution containing EDC, NHS, and antibodies was dropped at the BDDPE-WE and left for 24h at a temperature of 5 ^o^C. After incubation, the BDDPE was gently rinsed with demineralized water and dried. In the final step, the 50 µL of 0.1 mg/mL BSA solution was placed on the functionalized part of BDDPE and left for 1h at a temperature of 5 ^o^C. The sample prepared this way was used to detect protein D, Hi, and other bacteria.

**S.1.3. A protocol to perform the assay with an unknown sample**

The general protocol for specimen collection is specified by the Centers for Disease Control and Prevention or the World Health Organization (e.g. <https://cdn.who.int/media/docs/default-source/immunization/vpd_surveillance/vpd-surveillance-standards-publication/who-surveillancevaccinepreventable-05-haemophilusinfluenzae-r2.pdf>). To prepare the swabs for the electrochemical measurement 1 mL of it should be diluted in 1 mL of electrolyte utilized in the whole experiment (K_3_[Fe(CN)_6_] in 0.1 M PBS). A two-times higher concentration of ferrocyanide ions (10 mM) is required to obtain the same concentration as presented in the study, after dilution. The electrolyte (200 μL) should be placed on functionalized BDDPE and left for incubation (5 min). The EIS analysis is performed at -0.13 V vs BDDPE-RE pseudo-reference electrode. Before introducing such a protocol, it is essential to perform the negative control, which consists of the transport buffer and electrolyte without any biological material.

**S2. The electrolytic potential window of PE and BDDPE samples**

To assess the electrolytic potential window, the PE and BDDPE samples were deeply polarized in the range of hydrogen evolution reaction (HER) and oxygen evolution reaction (OER) and assess the electrolytic stability window. Although the Nernst equation describes the theoretical value of HER and OER potentials, the real values are usually different due to the presence of activation overpotentials. Furthermore, as small as possible non-Faradaic currents (resulting from electric double-layer charging) are desired within the electrolytic window. Any redox processes occurring at the electrode surface will be indicated by the presence of peaks in the voltammograms. **Figure S1** depicts the registered voltammograms.


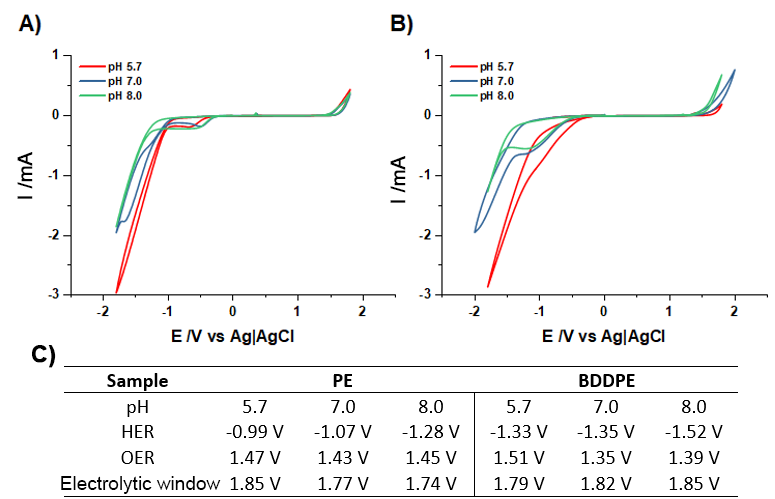


**Figure S1** – CV curves (3^rd^ cycle) recorded in phosphate buffer at different pH (5.7, 7.0, 8.0) for A) PE; B) BDDPE; C) Estimated HER and OER potentials at different pH. Scan rate ν = 100 mV/s.

Above a certain potential, the exponential current increase is associated with the OER. The exact value of the potential at which the increase of current appears is shown in **Fig. S1C.** The wide reduction peak is present below approx. -0.5 V and slightly changes with pH change. At more negative potentials, a sharp, exponential current drop indicates the start of HER during water electrolysis. In addition, **Fig. S1C** shows the electrode stability range, marked between the cathodic peak discussed earlier and the OER. As can be seen, the BDDPE samples overperform PE with increasing pH, but on the other hand, the visible reduction peak has higher values than in the case of PE samples. This may suggest that the BDD presence is acting as a catalyst for the PE reduction process causing its degradation.

**S3. Cyclic voltammetry curves registered for BDDPE and PE at different pH and scan rates**


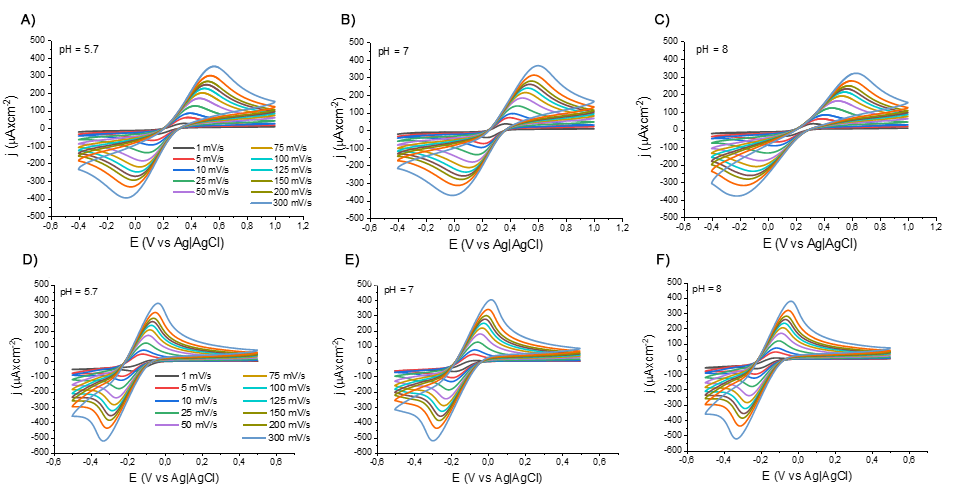


**Fig. S2 –** CV curves recorded for the PE samples in 5 mM K_3_[Fe(CN)_6_] (A,B,C) and in 5 mM [Ru(NH_3_)_6_]Cl_2_ (D,E,F) in PBS at different scan rates: (A,D) pH 5.7, (B,E) pH 7, (C,F) pH 8


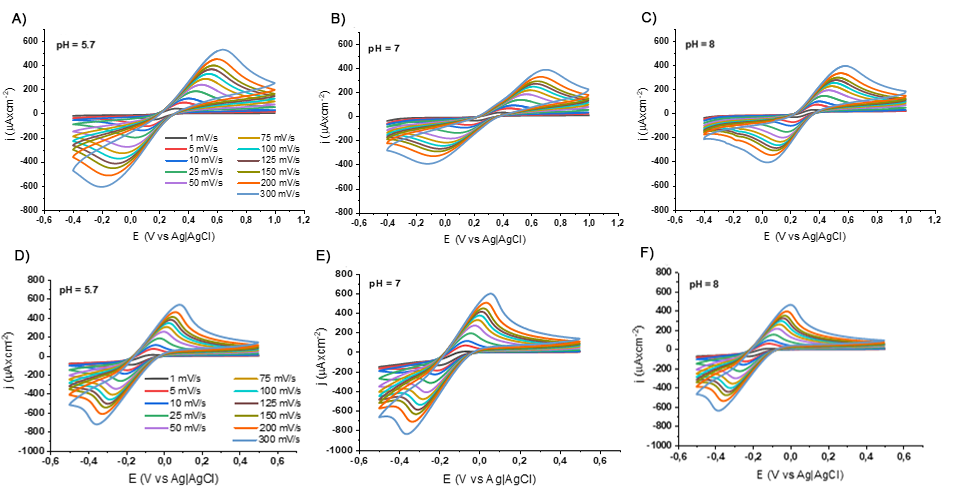


**Fig. S3 –** CV curves recorded for the BDDPE samples in 5 mM K_3_[Fe(CN)_6_] (A,B,C) and in 5 mM [Ru(NH_3_)_6_]Cl_2_ (D,E,F) in PBS at different scan rates: (A,D) pH 5.7, (B,E) pH 7, (C,F) pH 8


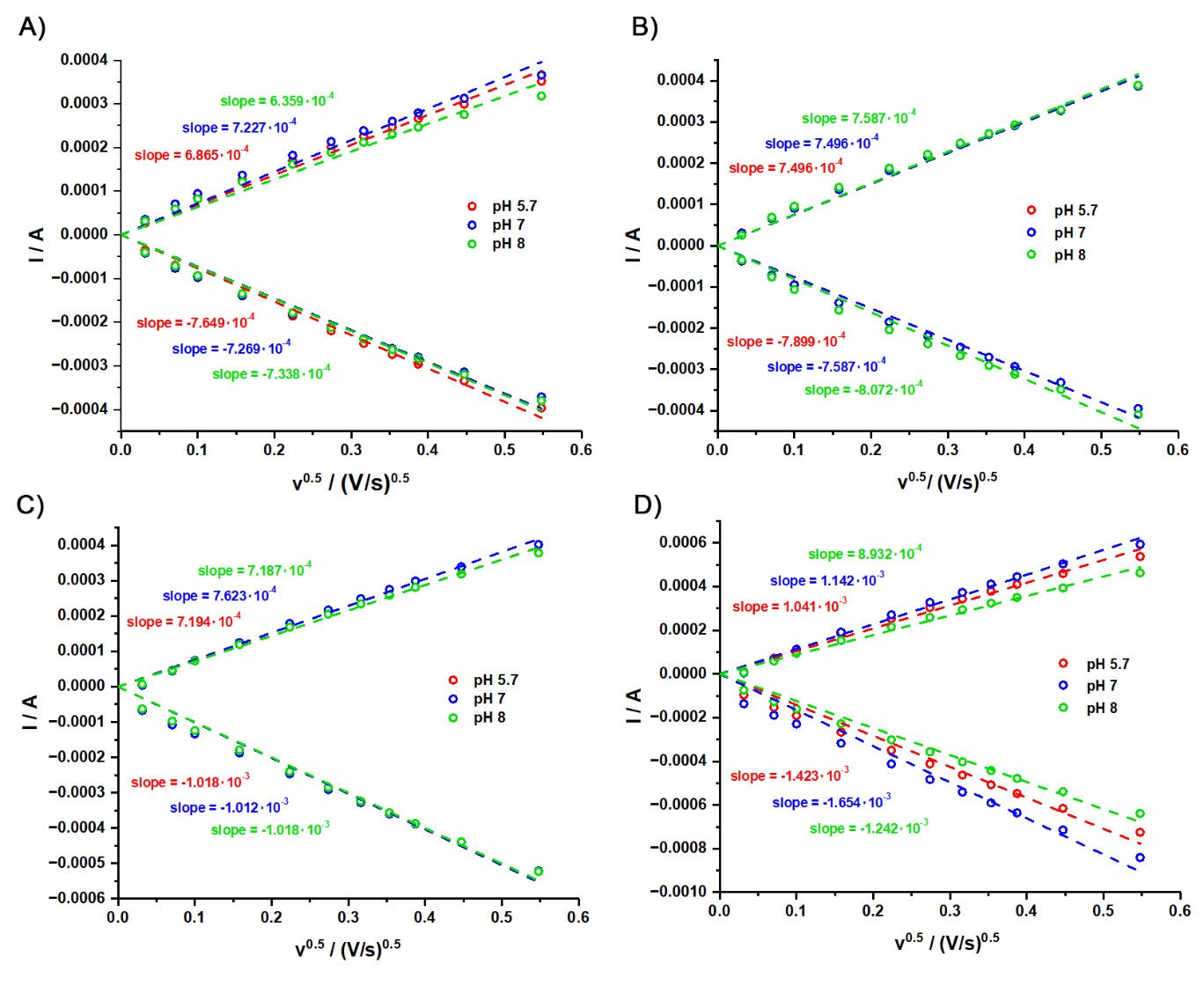


**Fig. S4 –** Plot of peak current density vs scan rate obtained based on CV curves for PE in A) K_3_[Fe(CN)_6_], B) [Ru(NH_3_)_6_]Cl_2_ and for BDDPE in C) K_3_[Fe(CN)_6_], D) [Ru(NH_3_)_6_]Cl_2_.

EASA was estimated using the following equation:

$i_{p}=2,99\cdot{10}^{5}\alpha^{\left( 1/2 \right)}AC_{O}D_{O}^{\left( 1/2 \right)}\upsilon^{\left( 1/2 \right)}$ (S1)

where: *i_p_* – peak current height [A], *α* – charge transfer coefficient (fixed at 0.5), *D_0_* – diffusion coefficient [cm^2^/s] (9.10۰10^-6^ cm^2^/s for [Ru(NH_3_)_6_]^2+/3+^ and 6.67۰10^-6^ cm^2^/s for [Fe(CN)_6_]^3-/4-^ [S1],
*C_0_* – concentration of redox species in the bulk of solution [mol/cm^3^] and *v* is a scan rate [V/s].

**S4. Supporting electrochemical impedance spectroscopy to the specificity studies**

**
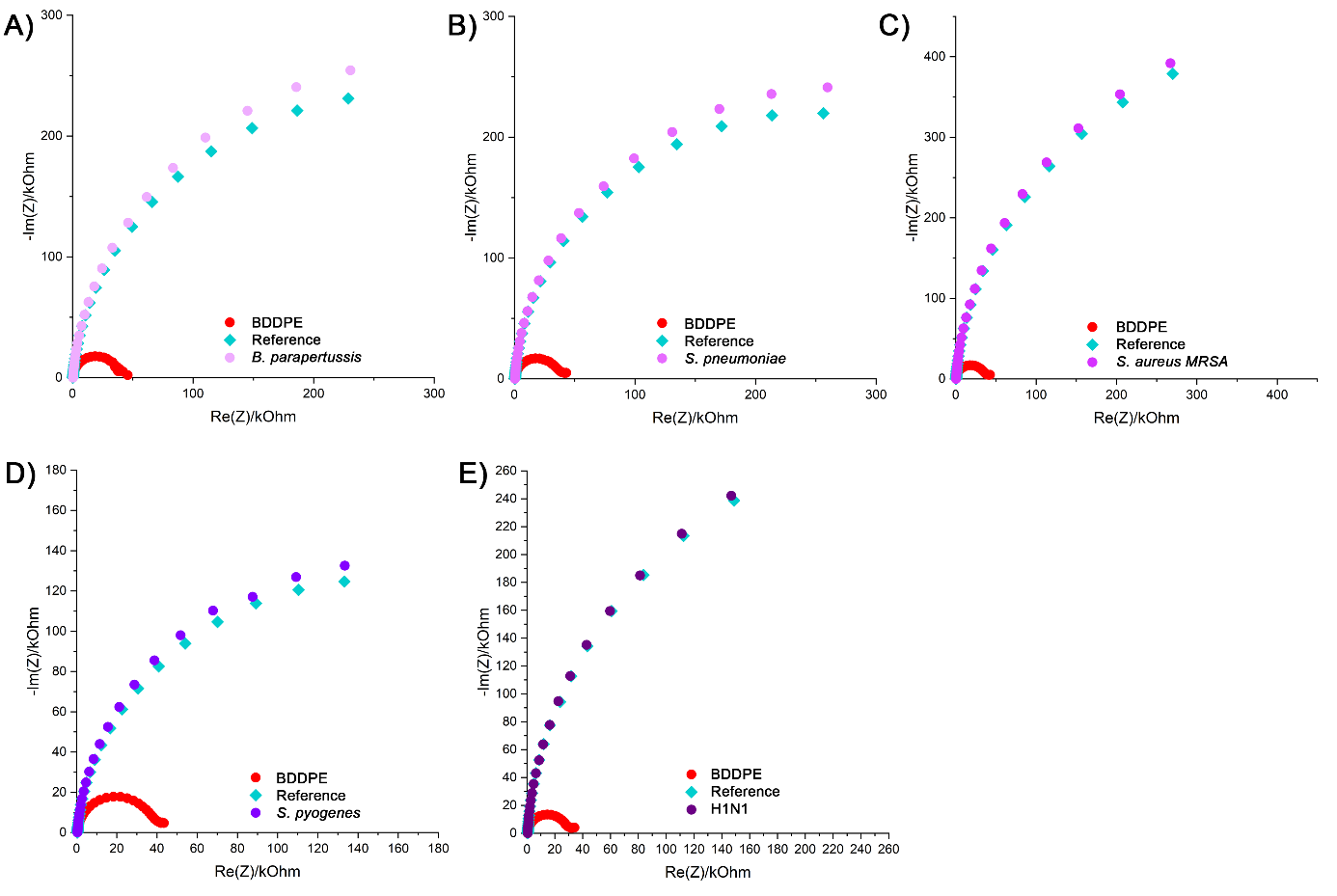
**

**Figure S5.** EIS Nyqust impedance plots for cross-reactivity tests studies with BDDPE. Frequency range 10 kHz – 1 Hz, measured at -0.13 V vs BDDPE-RE pseudo-reference electrode (E_F_) after 5 min incubation: Marking: BDDPE is unmodified electrode, Reference is BDDPE after functionalization.

**Table S2.** The EIS results calculated with the proposed EEC.

| **Pathogen** | **Concentration** | **CPE / µFs^n-1^** | **n / -** | **R_CT_ / kΩ** | **Response / %** |
| --- | --- | --- | --- | --- | --- |
| *B. parapertussis* | Blank | 1.67 | 0.95 | 483.9 | 10.73 |
|  | 10^5^ CFU/mL | 1.66 | 0.95 | 534.8 |  |
| *S. pneumoniae* | Blank | 1.51 | 0.95 | 463.5 | 9.34 |
|  | 10^6^ CFU/mL | 1.51 | 0.95 | 506.8 |  |
| *S aureus MRSA* | Blank | 1.38 | 0.95 | 874.4 | 6.04 |
|  | 10^6^ CFU/mL | 1.38 | 0.95 | 927.2 |  |
| *S. puogenes* | Blank | 1.68 | 0.92 | 282.3 | 7.90 |
|  | 10^6^ CFU/mL | 1.69 | 0.92 | 304.6 |  |
| H1N1 | Blank | 1.35 | 0.95 | 605.2 | 2.84 |
|  | 10^6^ CFU/mL | 1.33 | 0.95 | 622.4 |  |

**S5. References**

S1. Konopka SJ, McDuffie Bruce (1970) Diffusion coefficients of ferri- and ferrocyanide ions in aqueous media, using twin-electrode thin-layer electrochemistry. Anal Chem 42:1741–1746. https://doi.org/10.1021/ac50160a042

S2. Brodowski M, Kowalski M, Skwarecka M, et al (2021) Highly selective impedimetric determination of Haemophilus influenzae protein D using maze-like boron-doped carbon nanowall electrodes. Talanta 221:121623. https://doi.org/10.1016/j.talanta.2020.121623

S3. Jakóbczyk P, Kowalski M, Brodowski M, et al (2021) Low-power microwave-induced fabrication of functionalised few-layer black phosphorus electrodes: A novel route towards Haemophilus Influenzae pathogen biosensing devices. Applied Surface Science 539:148286. https://doi.org/10.1016/j.apsusc.2020.148286

S4. Sohrabi H, Majidi MR, Nami F, et al (2021) A novel engineered label-free Zn-based MOF/CMC/AuNPs electrochemical genosensor for highly sensitive determination of Haemophilus Influenzae in human plasma samples. Microchim Acta 188:100. https://doi.org/10.1007/s00604-021-04757-6

S5. Saadati A, Kholafazad kordasht H, Ehsani M, et al (2021) An innovative flexible and portable DNA based biodevice towards sensitive identification of Haemophilus influenzae bacterial genome: A new platform for the rapid and low cost recognition of pathogenic bacteria using point of care (POC) analysis. Microchemical Journal 169:106610. https://doi.org/10.1016/j.microc.2021.106610

S6. Saadati A, Hassanpour S, Hasanzadeh M, Shadjou N (2020) Binding of pDNA with cDNA using hybridization strategy towards monitoring of Haemophilus influenza genome in human plasma samples. International Journal of Biological Macromolecules 150:218–227. https://doi.org/10.1016/j.ijbiomac.2020.02.062

S7. Singh R, Hong S, Jang J (2017) Label-free Detection of Influenza Viruses using a Reduced Graphene Oxide-based Electrochemical Immunosensor Integrated with a Microfluidic Platform. Sci Rep 7:42771. https://doi.org/10.1038/srep42771

S8. Sohrabi H, Majidi MR, Asadpour-Zeynali K, et al (2023) Self-assembled monolayer-assisted label-free electrochemical genosensor for specific point-of-care determination of Haemophilus influenzae. Microchim Acta 190:112. https://doi.org/10.1007/s00604-023-05687-1

S9. Hassanpour S, Saadati A, Hasanzadeh M (2020) pDNA conjugated with citrate capped silver nanoparticles towards ultrasensitive bio-assay of haemophilus influenza in human biofluids: A novel optical biosensor. Journal of Pharmaceutical and Biomedical Analysis 180:113050. https://doi.org/10.1016/j.jpba.2019.113050

S10. Pickering JL, Prosser A, Corscadden KJ, et al (2016) Haemophilus haemolyticus Interaction with Host Cells Is Different to Nontypeable Haemophilus influenzae and Prevents NTHi Association with Epithelial Cells. Front Cell Infect Microbiol 6:. https://doi.org/10.3389/fcimb.2016.00050

S11. Abdeldaim GMK, Strålin K, Kirsebom LA, et al (2009) Detection of Haemophilus influenzae in respiratory secretions from pneumonia patients by quantitative real-time polymerase chain reaction. Diagnostic Microbiology and Infectious Disease 64:366–373. https://doi.org/10.1016/j.diagmicrobio.2009.03.030
